# Distributed Renewable Energy Management: A Gap Analysis and Proposed Blockchain-Based Architecture

**DOI:** 10.3390/jrfm15050191

**Published:** 2022-04-20

**Authors:** Annegret Henninger, Atefeh Mashatan

**Affiliations:** Ted Rogers School of Management, Ryerson University, 350 Victoria St., Toronto, ON M5B 2K3, Canada; amashatan@ryerson.ca

**Keywords:** blockchain, renewable energy, P2P, smart grid, microgrid, IT architecture, grid management, IT infrastructure, verifiable credentials (VC), decentralized IDs (DID)

## Abstract

The heterogeneous and decentralized nature of renewable energy sources is too much to handle for traditional and centralized IT grid infrastructure. Blockchain technology can address many of the associated challenges. This paper provides an overview of the state-of-the-art technology layers of grid system infrastructure, a proposed future state using blockchain technology, and gap analysis. The paper also contributes a set of architectural requirements for a blockchain-enabled future state and a proposed hybrid architecture using blockchain technology, verifiable credentials, and smart contracts. This architecture can uniquely support the technology layers critical to renewable energies, including system architecture, registries, grid management, billing, privacy, and interoperability.

## 1. Introduction

There are three categories of energy resources that supply global energy demand: fossil fuels, nuclear energy, and renewable energy. Fossil fuels and nuclear energy are reliable and cost effective. However, they produce significant greenhouse gasses and radioactive rays that contribute to global warming and climate change. Fossil fuels are also a finite resource approaching scarcity. Increasing consumption rates are causing an increase in the cost of carbon-based thermal power generation (Kåberger 2018). As a result, innovation practices largely focus on improving extraction methods and cutting costs. However, opportunities for technological improvements have been largely exhausted, and there is limited capacity for it to become less expensive in the future (Kåberger 2018). The economic pressures of the energy market and the growing concern for climate change have attracted researchers and innovators to the topic of renewable energy to help make it a competitive alternative.

Solar energy has developed rapidly as a result of having no pollutant emissions in the power generation process and no regional restrictions on resource availability (Wang et al. 2021). It is listed as one of the most successful renewable energy technologies and one of the only technologies being developed and implemented fast enough to impact climate change (International Energy Agency 2017). Solar energy from photovoltaic (PV) cells, in particular, is rated the fastest-growing energy source on the planet and the least expensive in many areas. Globally, the cumulative PV capacity surpassed 500 GW at the end of 2018 (International Energy Agency 2019; Solar Power Europe 2019), and in the UK, three percent of homes (1 million) installed solar PV cells (Strielkowski et al. 2017). The cost of solar panel projects using first-generation technology (crystalline silicon) has reduced by over 90% in the past decade (Kåberger 2018).

Despite advancements in solar energy, significant hurdles need to be overcome in the journey towards carbon neutrality and fighting climate change with renewable energies. For instance, while the available energy from sunlight exceeds the global energy demand, sunlight does not reach the earth’s surface evenly or consistently (González et al. 2021). This increases the need for effective energy storage, distribution, and management solutions, which have contributed to the advancement of smart grid and microgrid solutions (González et al. 2021). However, the distributed and heterogeneous nature of renewable energy sources, smart grids, and microgrids requires technical support to integrate them into the grid effectively (Alladi et al. 2019). The future of renewable energy is dependent on our ability to unlock the current limitations.

Blockchain technology can address grid integration challenges and is currently used in several conceptual renewable energy projects (Wang et al. 2021; Andoni et al. 2019). Noteworthy projects and contributions include the POC by Imbault et al. (2017), the Brooklyn Microgrid (Brooklyn Microgrid n.d.), Overgrid’s proposed architecture (Croce et al. 2016), Pop et al.’s prototype (Pop et al. 2018), Li et al.’s proposed energy consortium blockchain platform (Li et al. 2017), Bankymoon’s smart meters (Smart Energy 2015), eMotorWerk’s JuiceNet (eMotorWerks 2017), Powerledger’s platform (Power Ledger 2019), and LO3 Energy’s Pando (LO3 Energy n.d.).

The technology replaces top-down control with distributed consensus, open-source philosophies, transparency, and community-based governance. These characteristics at the root of renewable energy systems could inspire further societal change, such as more sustainable and responsible energy and resource consumption choices (Andoni et al. 2019).

Before determining a blockchain-enabled future state of renewable energy distribution, the current state of practice needs to be evaluated. In reviewing the decentralized renewable energy management literature, we have found that the current state of practice and a gap analysis between the current and future state is missing. Therefore, we offer a review paper to fill this gap. A review paper is an academic article based on existing literature, much like a systematic literature review or survey (Concordia University n.d.). A summary and evaluation of the existing literature explain the current state of practice, knowledge, and progress of the topic (Kim and Ji 2018). This view of the literature provides insights as to how ideas turn into accepted knowledge (McMahan and McFarland 2021) and can identify gaps between the current state of practice and the state of the art (Kim and Ji 2018).

Additionally, we contribute the following:

- A novel future state of blockchain-enabled distributed renewable energy management;
- A gap analysis between the proposed future state and current state of practice;
- A set of architectural requirements that are needed to support the proposed future state;
- A proposed architecture that meets those requirements.

This paper is organized as follows. An extensive literature review in Section 2 provides a background of blockchain technology and a review of the present and proposed future states of grid infrastructure. The present and future states are based on the six main areas we have identified: (1) architectures, (2) registries, (3) grid management, (4) billing, (5) relevant privacy issues, and (6) interoperability. The literature review is concluded with a gap analysis. In Section 3, a set of architectural requirements that would support the proposed future state are identified. In Section 4, an architecture that could support those requirements is provided. The paper is summarized in Section 5.

## 2. Literature Review

This section first provides an overview of the technology discussed in this paper. We then identify six critical technology layers of renewable grid infrastructure, review their present state of the art, and provide a proposed future state that is blockchain-enabled.

### 2.1. Technology Overview

This section provides a background on blockchain technology, decentralized identities, and verifiable credentials.

#### 2.1.1. Blockchain Technology

Blockchain and distributed ledger technology (DLT) are often used interchangeably. Both refer to a decentralized database with various distributed actors and nodes (Saurabh et al. 2020). This eliminates the single point of failure that leaves centralized systems vulnerable to malicious attacks and technical failures (Lesavre et al. 2019). Unlike centralized systems, every participant holds a copy of the ledger or can access it on the cloud, giving everyone in the network access to the historical log of transactions (Andoni et al. 2019). This allows all participants to verify their validity for a high level of transparency (Andoni et al. 2019).

Unlike DLTs, blockchains are immutable and append-only, due to their structure. Blockchains hold data as digitally signed transactions in a chain of sequential blocks that are time-stamped and cryptographically linked with hash functions (Yaga et al. 2018). Hash functions are one-way functions making them tamper-evident; any change to the data hashed results in a different hash function output (Yaga et al. 2018). Blockchain technology also supports smart contracts, which can be defined as software written as executable code (Mollah et al. 2020). The executable code automates the processes defined in the smart contract, making them self-enforcing. The reliance on code to execute the contract creates a shift in trust from people to math, enabling multiple parties to collaborate without a third party (Lemieux 2017; Mollah et al. 2020; Saurabh et al. 2020). The term blockchain has also been used to describe algorithms, digital consensus architectures, cryptocurrency, or applications/domains developed on top of distributed architectures—though these instances do not reflect the full definition of blockchain (Mattila et al. 2016).

Blockchains can be categorized by their type of access: open and closed (Andoni et al. 2019). Open blockchains, also known as public blockchains, are open to the public. They can be further divided into permissioned and permissionless blockchains (Lemieux 2017). The first blockchain, Bitcoin, is permissionless, whereby anyone can read, write, and otherwise participate (Nakamoto 2008). Public-permissioned blockchains enable the same level of transparency. The public can read but cannot write to a public-permissioned blockchain, making it suitable for use cases such as ownership registries (Tholen et al. 2019).

Closed blockchains are not open to the public and can be further categorized as private or consortium. Closed blockchains can be private-permissioned where only a select group of participants have read permissions, and only the network operator has write permissions (Tholen et al. 2019). Consortium blockchains grant authorized participants access to read and/or write (Tholen et al. 2019).

Blockchain networks can exist on top of existing technology infrastructure in what is called an overlay. Blockchain network services are decoupled from the underlying infrastructure using encapsulation (a packet inside another packet). For example, Overgrid’s proposed architecture is a P2P virtual representation of the existing physical grid based on fully decentralized overlay systems. It uses the Gossip protocol for node communication and an average update scheme to collect information on demand and supply (Croce et al. 2016).

A 2016 survey conducted by the German Energy Agency looked at the views of energy decision makers and revealed that nearly 20% believe blockchain technology is a game-changer for energy suppliers (Burger et al. 2016). Transactional blockchain platforms can provide energy firms with operational cost reductions, increased efficiencies, automated processes, and reduced capital requirements (Grewal-Carr and Marshall 2016). They can also accelerate the development of peer-to-peer energy trading ecosystems and reduce energy costs in microgrids at the community level (Burger et al. 2016).

#### 2.1.2. Decentralized Identities (DIDs)

A DID is a globally unique identifier encoded using a Uniform Resource Name (URN) (Saint-Andre Filament and Klensin 2017). It contains a DID document that includes DID metadata, the public keys, available service endpoints, and authentication (Reed et al. 2020). DID documents are kept in a registry that provides methods for creating, reading, updating, and deactivating (CRUD) documents. The verifying party retrieves these documents from the registry to authenticate the DID holder, using a challenge–response protocol (Alzahrani 2020). Some projects such as Alzahrani’s networking-based registry for DIDs and VCs (Alzahrani 2020) and Hyperledger’s Indy project (Hyperledger Indy 2018) use registries where DIDs can be viewed as a public string that maps to information in a registry and the DID owner’s private information. Together, the public and private information are used to assert a claim made by the DID owner.

#### 2.1.3. Verifiable Credentials (VCs)

DIDs can be used with VCs to support decentralized registries, enabling users to present a claim about credentials that can be instantly verified. Like a DID, VCs use registries and provide a machine-readable, tamper-evident method for asserting a truth (Alzahrani 2020; Sporny et al. 2017). They are digitally evolved versions of a long-existing concept, credentials. Credentials are issued by users and verified by someone who trusts the issuer of the credential and therefore trusts the credential (Trust over IP (ToIP) 2017), e.g., a physical ID card, diploma, or badge. However, such credentials can be forged, and centralized registries fail to validate authenticity as they are not designed to communicate with other systems. For example, it can take up to two weeks to obtain a police report or university transcripts sent to a prospective employer, and these present only a static set of facts on the individual.

VCs are well outlined in the W3C VC group’s VCs Data Model 1.0 (Sporny et al. 2017). They define VCs as data sets composed of claim data (payloads), metadata, and proofs. The claim data is what is being sent between the two parties. The metadata keeps information on the issuer, the intended recipient, the time it was sent, the source system, the destination system, the date and time it was issued, and a hash of the claim data and cryptography data. Proofs are the digital signatures of each party used to authenticate the claim.

### 2.2. Technology Layers

We have identified six critical technology layers to renewable energy grid system infrastructure: architecture, registries, grid management, billing, privacy, and interoperability. The following is a review of the current state of these layers and a proposed blockchain-enabled future state.

#### 2.2.1. Architecture

Decentralization is a factor facing renewable energy distribution as the number of distributed, heterogeneous, renewable energy sources increase (Andoni et al. 2019). Microgrids and peer-to-peer energy (P2P) transactions are two distributed systems that have recently emerged to support the distribution of community-based renewable energy efforts (González et al. 2021; van Leeuwen et al. 2020).

##### P2P

P2P energy trading is a new power grid operation and distribution model where individuals can consume, generate, and share electricity from local renewable energy sources (Zhang et al. 2016). P2P transactions are direct transactions between producers-cum-consumers (prosumers), who operate PV cells or small wind turbines for personal energy consumption and redistribute it back into the grid (Andoni et al. 2019). P2P sharing contributes to the energy balance and reduces congestion on transmission and energy distribution lines (Noor et al. 2018; Zhang et al. 2016). P2P schemes can also save money for energy consumers, generate revenues for prosumers and producers, reduce transmission losses, and promote the production and use of renewable energy (Noor et al. 2018).

The P2P market must manage the challenges that face the main grid, such as real-time price fluctuations, dynamically adjusted energy consumption, and frequent settlements (Noor et al. 2018). A low-cost robust energy trading system is needed to handle the energy market’s complex and dynamic information flows. P2P research is focused on determining the most appropriate technology to use in P2P trading (Park and Yong 2017).

P2P electricity trading has been growing despite these challenges, especially in deregulated areas (Park and Yong 2017). The world’s first online market for direct energy purchase opened in the Netherlands in 2014 with a startup, Vandebron. The Brooklyn Microgrid is an ambitious New York City proposal for blockchain-based P2P energy trading. The platform will enable prosumers to sell their energy surplus directly to their community using Ethereum-based smart contracts (Brooklyn Microgrid n.d.). It is powered by LO3 Energy. Other direct energy trading projects include UK’s Piclo, Germany’s SonnenCommunity, and China’s Energy Internet (Soto et al. 2020). P2P electricity trading without the need for utilities is expected to grow with P2P and microgrid awareness and accelerate with the advancement of renewable energy and internet technologies (Park and Yong 2017).

##### Microgrids

A microgrid is a local energy system consisting of distributed energy sources and demand capable of operating in parallel with, or independently from, the main power grid (Sujil and Kumar 2017; Yan et al. 2017). Microgrids should be able to run in isolation and integrate into the main grid to maintain a stable power source in the event of failure (González et al. 2021). A key feature of microgrids is their ability to separate from larger systems for unscheduled periods to support the microgrid (del Carpio-Huayllas et al. 2012). One of the benefits of microgrids is that they can reduce the distance between each power source and its user base (Noor et al. 2018). This translates to reduced transmission losses that occur when electrical energy is distributed from one point to another (Noor et al. 2018).

Microgrids can significantly benefit developing countries and less developed regions where the rapid urbanization and improved quality of life create increasing energy supply–demand stress (Cranston and Hammond 2010; Noor et al. 2018). In 2015, more than 10,000 isolated microgrids were operating in countries such as Bangladesh, China, and India (IRENA n.d.). Over the next ten years, a 20% increase in demand is forecasted for these regions (Government Publications Office 2016). Despite decarbonization and competitive energy pricing advantages, connecting microgrids and integrating renewable sources remains untapped in many areas. It is estimated to be a growing market worth over USD 200 billion annually (IRENA n.d.).

A popular microgrid architecture being explored is the honeycomb architecture, which represents the integration of individual microgrids (Imbault et al. 2017). However, with several interconnected networks, transaction security becomes a concern (Imbault et al. 2017).

##### The Blockchain-Enabled Future State of Architecture

A distributed system can facilitate P2P energy transactions and microgrids through smart-microgrid architectures (Wang et al. 2021). Smart grids are electricity networks that autonomously integrate the actions of all actors and objects connected to them to maintain reliable, efficient, and economical electricity supplies (Mollah et al. 2020; Zhang et al. 2016). Smart grids can be blockchain-based or use centralized technologies (González et al. 2021). However, a distributed energy system is more resistant to shortages or operational issues and can supply power to the grid seamlessly if a source is down (Andoni et al. 2019; Pop et al. 2018).

A decentralized approach can reduce the number of messages exchanged by circumventing a central authority, which jeopardizes the system’s availability and reliability (Pop et al. 2018). Blockchain technology is designed to enable decentralized transactions and remove centralized authorities (Grewal-Carr and Marshall 2016). For instance, Pop et al. developed and tested a blockchain-based grid management prototype using consensus to validate and activate appropriate financial settlements. The prototype performed near-to-real time event validation and financial settlements, and high demand–response signal accuracy (Pop et al. 2018). For an extensive list of blockchain-based smart grid solutions, see Mollah et al.’s survey (Mollah et al. 2020).

While there is strong support for using blockchain to support P2P networks and microgrids, there is no consensus on the most suitable type of blockchain. The variety of blockchain solutions provides benefits and drawbacks that need to be weighed when determining the best type of blockchain for a given application.

Public, trustless blockchain architectures for renewable energies could scale to a large size and provide transparency, making them censorship resistant. However, they will likely require high-cost consensus strategies and be slow to reach finality (finality is the assurance that the blocks committed to the blockchain cannot be altered). A large overarching blockchain also introduces risks of propagation delays across the network, with a consequential difference between the time a block is approved and when it is made available to the entire network (Vukolić 2015). Furthermore, slow finality and propagation delays introduce security risks, creating windows for forks and double-spending attacks (Vukolić 2015). Private blockchains could use lighter consensus mechanisms but may not scale to integrate an extensive energy network with many participants (Andoni et al. 2019).

**Figure 1:**
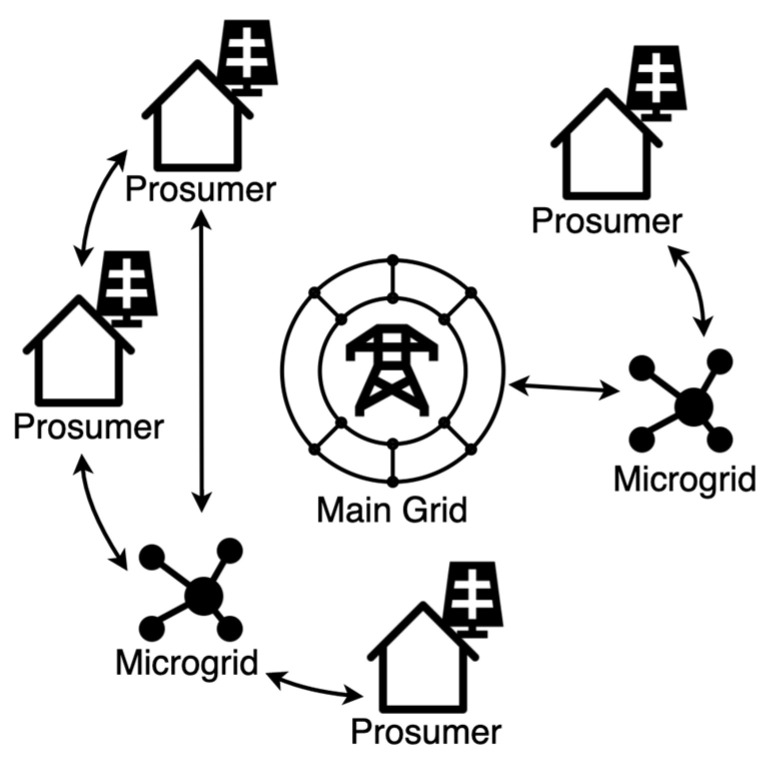
Hybrid Approach to Energy Distribution.

We believe a hybrid approach should be considered, integrating private microgrids and P2P energy trading into a more extensive public network, as illustrated in Figure 1. This way, networks that require instant, accurate information can run on smaller, private networks with lighter consensus mechanisms. Meanwhile, private blockchain-based microgrids connect to more extensive overarching networks that trust the authenticity and integrity of the microgrid.

#### 2.2.2. Registries

Registries are databases of information used across industries, e.g., registries for beneficial ownership, property, and business licenses. Important registries for the renewable energies market include green certificates, emissions certificates, and maintenance registries.

Green certificates act as a commodity product, proving that the associated electricity source is a renewable energy source (Imbault et al. 2017). Linares et al. found that the introduction of green certificates reduces the amount of required non-renewable energy and demand elasticity (Linares et al. 2008). Green certificates are stored in national registries for Guarantees of Origin (GoO) that provide all information concerning a given energy source (Imbault et al. 2017). Green certificates are a popular area of research in the literature, mainly focusing on their impact on efficiency and risk in the electricity market (Feng et al. 2018).

Carbon emissions allowances fall under carbon trading, which encompasses emission trading schemes for CO_2_ and other greenhouse gases in a market with limited carbon emissions allowances (Elkins and Baker 2001; Park and Yong 2017). Most developed countries that have implemented carbon trading also have capital subsidies, tax reductions (VAT), and some form of public investment (loans or grants) to promote small sources of renewable electricity (Park and Yong 2017). There is consensus in the literature that market-based carbon reduction strategies will reduce greenhouse gas emissions at a lower cost than regulation (Elkins and Baker 2001).

Maintenance registries are also crucial as they are an indicator of the health index of the equipment. This information is needed to prevent operational problems, predict costs, and determine the reliability of an energy source contributing to a microgrid (Imbault et al. 2017).

Most renewable energy sources and their energy storage systems are on microgrids. Unfortunately, the existing architectures for microgrids with decentralized assets of below 1 MWh do not presently support certificates (Imbault et al. 2017). There have also been security issues with such registries, including VAT frauds and quota thefts (Imbault et al. 2017).

##### The Blockchain-Enabled Future State of Registries

Blockchain technology could manage registries and make them available or transparent when needed (Tobin et al. 2017) can handle the increasing regulatory demands concerning energy sources and certificates (Tobin et al. 2017). Additionally, the transparency and traceability capabilities of the technology make it a suitable solution for the security issues of registries.

Two technologies that support registries and assume an immutable decentralized registry, such as a blockchain or P2P network, are decentralized identifiers (DIDs) and verifiable credentials (VCs). These use zero-knowledge proofs (ZKPs), an active area of research that allows a party to prove information about data without revealing the data (Saurabh et al. 2020; Wang et al. 2021). If incorporated into smart grids, registries could also be supported by the network diagnostics of an intelligent network. For instance, diagnostics for equipment maintenance and grid infrastructure could indicate a potential problem before it manifests and causes delays in the network (Alladi et al. 2019).

#### 2.2.3. Grid Management

The shift towards renewable energy sources presents new challenges to traditional grid management, particularly load management, flexibility, and information communication.

Load management in a decentralized heterogeneous network is more complex than in the traditional environment (Wang et al. 2011). Several renewable energy sources are needed to replace a single energy source from fossil fuels or nuclear energy. However, managing many heterogeneous energy sources and energy storage systems (ESSs) adds operational challenges because the power supply is intermittent, fluctuating, unpredictable, and out of sync, making it difficult for utilities to predict the energy supply (Alladi et al. 2019; Blarke and Lund 2008; Imbault et al. 2017).

Flexibility is a critical issue in shifting to a dependency on renewable energy. While grid operators can handle price fluctuation in traditional or small-scale renewable energy grids, large penetration necessitates increased operational flexibility of the energy system (Blarke and Lund 2008). Flexibility becomes a challenge with uneven, rapid demand growth (Noor et al. 2018).

There is also limited flexibility on the consumer side, due to a lack of mobility in traditional energy markets. Consumers sign up with an energy provider and have little control over where their energy comes from (Burger et al. 2016). A UK government report revealed that poorly designed tariff prices and a lack of flexibility have contributed to electricity consumers paying an excess of GBP 1.4 billion on average per year in the period 2012–2015 (Competition and Markets Authority 2016). 

Traditional information communication technologies (ICTs) also struggle to manage energy markets’ increasing digitization and decentralization (Andoni et al. 2019). For instance, many countries have feed-in-tariffs enabling individuals to sell energy back to the main grid at a fixed price (van Leeuwen et al. 2020). However, renewable distributed energy resources are often situated in the fringes of low voltage (LV) distribution grids where the technology infrastructure is not sophisticated enough to handle numerous heterogeneous energy sources (van Leeuwen et al. 2020).

##### The Blockchain-Enabled Future State of Grid Management

Blockchain technology can aid grid management, enabling many different parties to connect and interact securely. It can manage varying and growing energy demands, integrating P2P transactions, distributed LV sources, ESSs, and communication between distributed parties. By incorporating smart contracts, a blockchain-based smart grid could respond quickly and autonomously to energy supply and demand changes and effectively manage ESSs.

Regarding flexibility, blockchain-enabled decentralization allows for greater ease of mobility in energy markets, which helps increase flexibility (Burger et al. 2016). Consumers could stipulate the maximum rates they want to pay and percentage thresholds for the energy they are willing to consume from non-renewable sources (e.g., consuming a maximum of 30% of carbon-based energy). Consumers could also prioritize buying or selling energy from certain providers, e.g., a renewable energy provider they want to support or friends/family/neighbors (Mengelkamp et al. 2018). Furthermore, users could change their preferences at any time. Such mobility in the energy market mimics the flexibility and integration of thresholds seen in stock markets (Andoni et al. 2019). Increasing flexibility and consumer power could increase competition in renewable energy markets (Burger et al. 2016).

Regarding communication, blockchain-based frameworks can support grid management by leveraging wireless sensor networks and IoT to better monitor usage (Alladi et al. 2019). Integrating advanced metering devices can increase the accuracy of usage statistics and lower labor costs (Alladi et al. 2019). Pop et al. designed and tested a prototype of a blockchain-based system that collected prosumption information from IoT smart metering devices and stored it on the blockchain (Pop et al. 2018). The authors used smart contracts to define the energy flexibility of each prosumer, the related rewards or penalties, and the coded rules for balancing demand and supply at the grid level (Pop et al. 2018). Their prototype successfully matched demand and production at the smart grid level, with high accuracy of demand–response signals and a reduced need for energy flexibility for convergence.

Regarding load management, Pop et al. found that the rapid increase in distributed energy prosumers (DEPs) makes centralized approaches no longer suitable to manage smart grids (Pop et al. 2018). The authors also found that the rise in renewable energy sources and IoT smart metering devices has driven decentralized grid adoption beyond grid-scale energy storage capacities. A lack of surplus plus the intermittent and unpredictable nature of renewable energies on the grid can lead to deficits and overload, causing power outages (Sujil and Kumar 2017).

Distributed system operators can decrease the output from energy sources to protect the grid (Pop et al. 2018). However, a better approach would be to focus on demand-side management: matching demand with production by motivating DEPs (Han et al. 2015). The demand system operator communicates a demand response event to the DEPs at the start of a billing period. DEPs are incentivized to modify their consumption and respond with a bid containing the amount they propose to decrease to or increase to (Han et al. 2015; Kyriakarakos et al. 2013). Smart contracts pre-programmed with existing thresholds would enable the process to be completely automated. Studies have applied a hierarchical framework (X. Jin et al. 2017), mixed-integer linear programming (MILP) (Kriett and Salani 2012), evolutionary algorithms (Onyeji-Nwogu et al. 2017), dynamic programming (Livengood and Larson 2009), game theory (Prete and Hobbs 2016), multi-objective optimization (M. Jin et al. 2017) and several different modeling approaches (Han et al. 2015; Nguyen et al. 2014). 

#### 2.2.4. Billing

Renewable energy networks need to manage many of the billing challenges facing traditional main grids, such as handling third parties (Noor et al. 2018), transparency and auditing (Andoni et al. 2019), and managing frequent settlements (Noor et al. 2018).

Managing third parties is difficult in wholesale energy markets. They can be complex and opaque, consisting of complicated processes requiring several intermediaries, including trading agents, brokers, logistics providers, exchanges, price reporters, and regulators (Andoni et al. 2019). Third parties significantly increase the cost of operations and mistakenly or intentionally allow for opportunities for erroneous transactions (Alladi et al. 2019). As a result, transparency and auditing amongst the participants are imperative.

Managing frequent settlements is a complex process. An operator can schedule or manage the energy output and output pricing (Ela et al. 2016). For example, in the USA, all scheduled market regions use 5 min scheduled output intervals (Ela et al. 2016). However, while some resources are paid for based on the individual 5 min prices, most are based on the hourly average (Ela et al. 2016). Frequent and short intervals enable more accurate pricing, providing significant incentives for consumers or distributors to follow and respond to pricing fluctuations (Ahl et al. 2019). However, this is only performed in select sophisticated markets, including Southwest Power Pool and New York Independent System Operator (NYISO) (Ela et al. 2016). For energy wholesalers, reconciliation delays are a significant issue, due to lengthy reconciliations, confirmations, and volume actualization (Grewal-Carr and Marshall 2016).

##### The Blockchain-Enabled Future State of Billing

Blockchain technology is particularly adept at handling billing and transaction processes. It was initially designed to address the double-spend problem to secure online transactions and has been extensively used in payment processing (Nakamoto 2008).

Blockchain technology can remove third-party issues by enabling participants to transact directly per predetermined conditions stated in the smart contract (Alladi et al. 2019). It can record a tamper-evident history of bids and offers with automatic order fulfillment execution. The tamper-evident records in blockchain technology also enable effective product provisioning by providing a single point of truth for information such as historical energy consumption (Grewal-Carr and Marshall 2016). From a social impact perspective, exposing consumers to the dynamic cost of energy generation may encourage more sustainable energy consumption and appropriate price signals in response to demand.

Blockchain-enabled transparency and auditing functions support effective price discovery, compliance and auditing requirements, logistics, margin, know-your-customer (KYC), reconciliation, financial reporting, and settlements (Mercier and Yu 2017). For example, Pando is a platform used to launch a community energy trading marketplace. It includes a configurable marketplace platform, a mobile app, and a portal that shows demand fluctuations. Users can make offers, budgets and sell surplus energy on the platform (LO3 Energy n.d.). However, despite blockchain’s transparency and auditability capabilities, information on a blockchain does not necessarily reflect real-world events; there is no safeguard to guarantee the accuracy of data recorded on the blockchain. At the same time, the more events are recorded on the blockchain or integrated from another chain, the more reliable the information is. Reducing alterable, paper-based processes in the network reduces fraud or human error opportunities.

Blockchain-based energy billing solutions also provide more payment options than traditional systems. Many solutions incorporate virtual currencies or credit-based transaction systems that enable participants to purchase energy without possessing the currency at the transaction time (Li et al. 2017). For example, Bankymoon is a South Africa-based startup developing smart meters with integrated payments that accept cryptocurrencies (Smart Energy 2015). A survey of energy-based cryptocurrency initiatives can be found in Mollah et al.’s work (Mollah et al. 2020). A credit-based payment scheme would allow users who do not possess enough coins to purchase energy on credit (Alladi et al. 2019). For example, Li et al. proposed a credit-based system that offers a pricing strategy based on a Stackerlerg game for credit-based loans for participants with insufficient energy coins (Li et al. 2017). Their system supports fast and frequent energy transactions and uses energy aggregators to store and manage them.

In theory, automation enabled by smart contracts would execute regular real-time settlement pricing (Ahl et al. 2019). Streamlined, transparent, blockchain-enabled processes could support near-to-real time confirmations and volume actualization and reduce the need for reconciliation. For example, Share & Charge enables P2P energy trading for EV private charging (eMotorWerks 2017). E-wallets provide users with real-time prices and process transactions on the Ethereum-based platform. Billing and certificates are automatically processed (eMotorWerks 2017). In an energy imbalance, blockchain-enabled smart contracts could identify precisely which power source caused the imbalance and automatically make adjustments.

A significant challenge facing the application of decentralized technologies in decentralized energy billing is their inability to handle fast and frequent transactions (Wang et al. 2021). Bitcoin, for instance, can process approximately seven transactions per second, validating a block every 10 min, with each block containing a few thousand transactions. However, it takes approximately 1 h to reach finality (Buchko 2017). Early blockchains developed using Ethereum could process 20 transactions per second (Kramer and Hartnett 2018).

Consensus mechanisms are widely seen as the bottleneck for creating a new block on a blockchain as they control the scalability, speed, and security of transactions. Several new consensus mechanisms have been proposed and can be classified as lottery-based or voting-based (Andoni et al. 2019). Lottery-based systems are less adept at scalability as they may result in multiple chains that need to be resolved to reach finality. Voting-based consensus mechanisms achieve finality quickly but may take longer to reach a consensus (Andoni et al. 2019).

Lottery-based approaches include the proof of work (PoW) and proof of stake (PoS) mechanisms. The most widely used lottery-based approach, PoW, rewards nodes that solve cryptographic problems to validate transactions and create a new block on the blockchain (Andoni et al. 2019). PoS systems have validators selected at random or through a round-robin scheme and whose weight of their ‘vote’ is determined by their stake in the system (Andoni et al. 2019).

Meanwhile, voting-based mechanisms are based on the practical byzantine fault tolerance (PBFT) algorithm (Andoni et al. 2019). Several other mechanisms exist, including delegated proof of stake (DPoS), federated byzantine agreement (FBA), proof of authority (PoAu), proof of activity (PoAC), proof of elapsed time (PoET), proof of burn (PoB), proof of capacity (PoC), proof of inclusion (PoI), proof of activity (PoAc), leased proof of stake (LPoS), delegated proof of stake (DPoS), and proof of elapsed time (PoET) (Mollah et al. 2020).

The finality and lag issues of the interconnection of blockchains, IT devices, and Dapps can become highly difficult to manage in more extensive networks. For instance, it is extremely difficult to retrieve and revert to previous transactions that depend on other transactions, and the number of dependencies can grow extensively in a dense network (Belchior et al. 2020).

#### 2.2.5. Privacy

The integration of smart devices, especially when integrated into centralized systems, poses a significant risk to privacy. Many consumer smart devices enable users to introduce energy-efficient settings and remotely control their home’s energy consumption. Components include social media networks, smart home devices (e.g., smart thermostats and smart cat food dispensers), wearable technology (e.g., smartwatches and step counters), and industrial and business applications (e.g., motion sensors and RFID readers). The point of IoT is that the devices are ubiquitous, enabling us always to be connected.

However, smart grids and smart devices also introduce privacy risks (Mollah et al. 2020). Smart energy systems and associated IoT devices handle and are linked to users’ personally identifiable information (PII) and sensitive information, including home address, account, billing information, and time-stamped, location-identifying information about their routine activities. This makes IoT networks an attractive target for malicious actors. Additionally, most IoT devices are small and cannot host robust cryptographic algorithms to protect the information, which risks the network.

Furthermore, centralized information systems have a single point of failure that poses privacy risks (Aitzhan and Svetinovic 2016; Pop et al. 2018). With all information flows going through a central authority, the system is vulnerable to a man-in-the-middle attack whereby a malicious party can collect patterns and daily routines of prosumers, as well as their locations collected from IoT devices in the smart grid (Aitzhan and Svetinovic 2016; Pop et al. 2018).

##### The Blockchain-Enabled Future State of Privacy

The benefits of IoT devices are amplified when integrated with blockchain technology; smart contracts can automatically take actions using the data generated from the smart device in near-to-real time (Mollah et al. 2020).

Additionally, blockchain technology can provide secure data transfer and storage for these devices. However, new privacy challenges arise when using blockchain technology.

From the wholesaler’s perspective, while the transparency capabilities of blockchain technology can provide needed visibility in energy markets, energy wholesalers and competing intermediaries may not want to disclose competitive secrets and contacts to the network. Similarly, with individual and community-based sources of renewable energy being incorporated into the primary grid, the privacy of those individuals needs to be protected in compliance with the strict regulations introduced by GDPR (Tholen et al. 2019). This vulnerability amplifies human-error-based risk; a participant may accidentally publish sensitive information on the blockchain which cannot be removed.

Research on solutions for blockchain privacy is an active area of research with many solutions at the forefront of technology, including DIDs, VCs, centralized and decentralized mixing services, homomorphic and semi-homomorphic encryption, ring signatures, and group signatures.

DIDs and VCs are gaining attention with regard to managing privacy as they increase users’ control over their data. DIDs and VCs also enable proof of existence (PoE) capabilities, allowing participants to prove something is true without revealing the information. Blockchain can support DIDs with built-in credentials and control mechanisms and support the scalability of identifiers at a low cost (Sporny et al. 2017). The scalability of DIDs is essential, as both actors and objects across the entire network will need identifiers.

Another proposed solution is a mixing service. A mixing service acts as a relay node, encrypting and decrypting messages with the mixing services’ public key and directing it to the intended recipient. This process can obscure the messages, making mapping to the individual users more difficult. There are many centralized mixing services available, especially for the use of cryptocurrencies (e.g., Blindcoin, Mixcoin, Trumble, and Xim) (Bonneau et al. 2014; Heilman et al. 2017; Bissias et al. 2014). However, these are centralized and introduce a single point of failure at the relay node. Coinjoin is a decentralized mixing service; though it applies specifically to the Bitcoin blockchain and its ability to have several output addresses, it is not necessarily applicable to other blockchains.

Another solution is homomorphic encryption, a public-key encryption scheme that can be traced back to the seminal work of Rivest et al. (1978) where it was first referred to as privacy homomorphism. It enables us to calculate linear functions of an encrypted input using only the ciphertexts (Asharov et al. 2012). However, it adds a significant computational overhead, which has led to the investigation into alternatives such as semi-homomorphic encryption (Bendlin et al. 2011).

Semi-homomorphic encryption allows plaintext to be retrieved as long as there is a minimal increase in the input size (Bendlin et al. 2011). It can be used in multiparty computation (MPC) as well. MPC enables multiple participants to preserve their privacy while contributing their encrypted input to the computing function (Asharov et al. 2012). The inputs are never revealed in unencrypted form and are used to obtain the combined score. However, it must be protected against a dishonest majority, which is challenging to manage efficiently, and must use public-key technology (Bendlin et al. 2011).

Ring signatures are another tool used to mask the mapping of addresses. Ring signatures introduce signer ambiguity, linkability, and unforgeability (Alonso 2018; Rivest et al. 2001). Initially, group signatures relied on a shared secret and were sometimes set up and managed by a central system (Chaum and Van Heyst 1991). This kind of coordination and secret sharing is not scalable and jeopardizes anonymity (Alonso 2018). Liu et al. (2004) proposed a type of group signature where the sender can produce an anonymous signature by choosing co-signers from a directory of candidate public keys without collaborating with them. When the message is broadcast, a third party verifies that one of the private keys (without knowing which) corresponds to the public key and is used to sign the message (Alonso 2018). Monero is a well-known blockchain that uses this privacy-preserving ring signature (Alonso 2018).

#### 2.2.6. Interoperability

Interoperability is a challenge facing all technology (Liu et al. 2019). Henninger and Mashatan (2021) describe enterprises in the current, interoperable state as digital islands, each with their digital capabilities, not designed to and unable to integrate with other systems. Regardless of digital sophistication, each enterprise’s capabilities are limited to whatever systems or data exist within their boundaries. With the rise in renewable, distributed, heterogeneous energy sources, interoperability is becoming a larger issue facing energy systems.

##### The Blockchain-Enabled Future of Interoperability

Interoperability is an inherent problem in blockchain (Liu et al. 2019). Most research explores this issue from a technical angle as it is the key to unlocking the scalability of blockchain business applications. Most solutions propose validators to connect blockchain networks (Koens and Poll 2019). Current notable efforts and projects include Polkadot, Cosmos Network, Wanchain, HyperLedger Cactus, and standardization efforts.

Polkadot is a multi-chain framework designed to advance the interoperability and scalability of blockchains in the ‘relay network’ (Deng et al. 2018). The framework connects ‘parachains’, which can be blockchains or other data structures, and the relay chain can bridge heterogeneous chains using different consensus mechanisms together (Deng et al. 2018). Validators finalize blocks and run the entire relay chain application with a deterministic selection phase and ratification. They may also elect other validators to run in their place.

The Cosmos Network aims to solve scalability and interoperability problems by creating an internet of blockchains. The blockchains are attached to a central ledger where individuals keep their tokens (Deng et al. 2018). However, this introduces a fundamentally centralized structure incongruent with the decentralized nature of distributed networks.

Wanchain is a universal cross-chain protocol that connects and transfers value between heterogeneous blockchain ledgers, focusing on financial services, i.e., cryptocurrencies (Deng et al. 2018). Validators secure the central ‘hub’ in the Cosmos structure; they verify transaction correctness and agree on the block to be committed to the chain. If the block comes from a sovereign blockchain connected to the hub, the cosmos validator set will simply change the state (Wanchain n.d.). Smart contracts lock tokens on the issuing blockchain and create equivalent tokens on the receiving blockchain (Wanchain n.d.).

HyperLedger Cactus (previously the Blockchain Integration Framework) is a tool for integrating blockchains (Belchior et al. 2020). It has a pluggable architecture that enables operations to work across heterogeneous blockchains (Belchior et al. 2020). It proposes to use interoperability validators from the source and target blockchains to validate the transactions between chains (Belchior et al. 2020). Currently, the project supports integration with HyperLedger frameworks (Belchior et al. 2020).

Another key component of interoperability is standardization. The European Committee for Standardization (CEN), European Committee for Electrotechnical Standardization (CENELEC), and European Telecommunications Standards Institute (ETSI) proposed a framework for smart grid architecture models (SGAM) to enable European standardization organizations to support the continuous development of standards for smart grids (Baliga 2017). The SGAM framework has four layers: (1) the power grid layer, which includes existing electrical grids, ESS, EVs, flexible loads for managing demand and supply, etc.; (2) the ICT layer, which supports communication between devices and data storage; (3) the control layer, which monitors the integrated systems and supports control functions; and (4) the business layer which supports energy trading, storage services, and intermediary services.

Blockchain technology and smart contracts can support layers 2–4. By providing a single point of truth, blockchain technology supports cross-boundary processes enabling several stakeholders to connect and collaborate on each layer and support smooth information flows across the layers.

### 2.3. Gaps

While the future for renewable energies is bright, it faces many challenges. Main gaps can be grouped under architecture, scalability, privacy, interoperability, and governance.

#### 2.3.1. Architecture Gaps

The literature identifies several gaps facing blockchain-based energy grid architectures, including the following.

- *Communication*: the integration of prosumer-heavy microgrids into the P2P energy economy adds complex communication challenges (Park and Yong 2017; Wang et al. 2021). Every node needs to respond to changes in supply and demand, prices, and grid conditions. While this may be feasible with smaller markets, a global network of integrated microgrids could not manage timely propagation across all the nodes. Therefore, an architecture for a network of microgrids with different boundaries of operation needs to be determined.
- *Operational challenges*: Noor et al. found that microgrid models are flawed in their assumption of sufficient energy supply (Noor et al. 2018). Operational challenges such as the intermittent and uncertain nature of renewable resources, resource seasonality, storage, conversion, and distribution make it difficult for a honeycomb architecture of renewable resources to provide reliable, secure energy when not connected to the main grid (Noor et al. 2018; Sujil and Kumar 2017). These issues are particularly applicable in the energy markets of the global south, characterized by supply shortfalls and load shedding (Noor et al. 2018). More solutions to managing the energy supply, demand, and storage of microgrid architectures are needed.
- *Design*: what the public has a right to know regarding the source and distribution of their renewable energy needs to be determined. This gap needs to be addressed from a managerial, community, and public policy perspective.
- *Type of Blockchain*: frameworks that identify which type of blockchain is suitable in which circumstance would provide a foundation to support future research on the topic.

#### 2.3.2. Scalability

Scalability is a highly cited concern with blockchain technology, often on the premise of limited throughput (McMahan and McFarland 2021; Mollah et al. 2020). Efforts in increasing scalability and speed include developing new consensus algorithms, sharding, side chains, pruning, and growing block sizes. However, blockchain technology is still far from reaching the transactional speed of traditional technologies, such as Visa’s system, which supports 24,000 transactions per second (Andoni et al. 2019). An architecture of consensus mechanisms that can maintain low propagation delays and support fast and frequent transactions where required needs to be determined (Mollah et al. 2020). Additionally, consensus algorithms must be resilient to node failures, corrupt messages, message lag, and unreliable or corrupt nodes (Andoni et al. 2019; Baliga 2017).

Consensus mechanisms will also need to align with the renewable energy market’s sustainability vision. The use of blockchain technology should not noticeably reduce the energy supply. Blockchain technology has been criticized for being computationally intensive and requiring significant power to run (McMahan and McFarland 2021; Fairley 2017). This criticism stems from the computational intensity of some consensus mechanisms, such as PoW, which is known to be energy-intensive and requires significant amounts of energy to validate transactions (McMahan and McFarland 2021). Lighter options have been developed, including PoS and voting-based mechanisms suitable for different use cases.

#### 2.3.3. Privacy

Cryptographic solutions need to be used to address privacy risks (Saurabh et al. 2020). Solutions mentioned in this paper include zero-knowledge proofs, multiparty computation, homomorphic encryption, and ring and group signatures. A recent notable framework for P2P energy management was proposed by Wang et al. in 2021 that addresses this issue. Their framework uses a permissioned blockchain with assigned unique IDs with entity mapping, and zero-knowledge-proofs to protect identities. The cryptographic protocols, data models, algorithms, and code would need to be open source to support the overarching decentralized objectives and be trusted. However, these technologies are conceptual or still in the early implementation phases, and the data structures for this implementation would need to be constructed.

Architectures that store less data on the chain could also be explored. Lesavre et al. argue that such architectures could be a more secure option to protect against new vulnerabilities discovered by cryptographic solutions or existing authentication and messaging protocols (Saurabh et al. 2020).

#### 2.3.4. Interoperability

The interoperability between different blockchains, and a blockchain and exiting internal systems remains a challenge (McMahan and McFarland 2021). This can lead to restricted data sharing, limited data access, and low integration of a blockchain-based solution (McMahan and McFarland 2021). While several advances have been made towards increasing interoperability, they introduce the problem of fragmented, tailored solutions addressing specific use cases, further complicating interoperability. There is a gap between theory and real-world application and practice; most proposals are conceptual (Belchior et al. 2020).

#### 2.3.5. Governance

There is no consistent standard or best practice of governance amongst blockchains and decentralized ledgers, let alone an overarching system of blockchains. Appropriate governance for decentralized energy grids must be determined to ensure grid quality and reliability (Belchior et al. 2020). Integrated P2P networks could introduce energy equipment overload, electrical energy quality degradation, forced load reduction, and shutdowns (Park and Yong 2017). Additionally, the governance needs to be flexible enough to function in a chaotic state, i.e., the manifestation of emergencies or exceptional circumstances that require exceptional decisions and handling (Reijers et al. 2018). Chaotic states include wars, pandemics, natural disasters, and financial crises. As this review is being written, we are in the middle of a global pandemic, COVID-19, that has changed many behavioral patterns and energy use (IEA 2020).

## 3. Requirements

The following section identifies the requirements for an improved future state of renewable energy distribution. The objective is to extend standard functionalities, interoperability, and trust across the various systems and transactions. Requirements are presented at four levels: framework, platform, architecture, and use case-specific.

The framework is defined as common artifacts that include standards, reusable components, and processes. Standards need to be applied to the glossary of terms, communication protocols, data models, data format specifications, and system design. Reusable components include established classes and class hierarchies, code, interface designs, libraries, etc. Processes include the governance methodology, platform administration, maintenance, provisioning, and sunsetting.

A platform instantiates the framework, acting as a reference implementation that commits to the framework to enable a use case. It maps the operating environment to the use case, has standardized use case vocabularies, and supports ontology for non-domain specific definitions.

The architecture describes the software requirements that meet the specifications and support customization within the use cases. It includes the descriptions of subsystems, data, and protocols. The use case here is the implementation of a smart grid for renewable energies.

The specific requirements are listed in Section 4 to show how our architecture satisfies each of them. Throughout the requirements, we notice three areas that need attention: identification, communication, and standardization. Many of the requirements indicate the need for unique identifiers to identify subjects across heterogeneous systems. A subject can be an individual, a system, organization, data, device, asset, task/action, role, etc. The original system identifier needs to be preserved, and cross-system identifiers need to be provided by a system. This must be done to prevent the duplication of subjects across systems.

The requirements also highlight the need for standardization, as highlighted in Mollah et al.’s survey (Mollah et al. 2020). This includes standards for communication, data structures, architectures, and protocol for subjects, their attributes, and the transactions in which they participate. Cross-system processes and information sharing depend on standardized ways of managing cross-system requirements, including auditing, identification of subjects, comparison/translation, bridge asset representations, and task representations.

The requirements also highlight a need for effective communication: there should be a straightforward way to identify and verify the sender, receiver, order, and contents. Such clarity should be extended to the systems and ties into standardization; systems need a standard way to locate and communicate with one another. Decentralized globally unique identifiers are needed for subjects and messages to communicate data.

## 4. Overarching System Architecture

In the blockchain-centric literature, proposing models, frameworks, architectures, and POCs are popular contributions which help to progress the technology. We find that the overarching architecture of such contributions can be divided into four approaches: network, centralized, decentralized, and VC-based.

In a network approach, each system is a peer that can communicate with other peers (Mödinger et al. 2018). For example, peer systems can map identifications and translate data between standards (Mödinger et al. 2018). This reduces overall complexity by removing the need for a meta-system or related system management and governance. However, a network approach requires all existing participating systems to update their system whenever new additions to the system need to be included. This risks the network’s systems being out of sync.

A centralized system depends on a singular system acting as a hub. The participating systems communicate through the central hub, which can handle standard translations and identifier mapping. However, this can create bottlenecks and a single point of failure (Mitrovic et al. 2016; Wang et al. 2021). Additionally, a centralized hub does not have the transparency capabilities of a decentralized system to protect against collusion and to provide traceability. Transparency and traceability of transactions are essential for participants to trust or verify that the network is fair and without collusion. A centralized system approach would require trust that there is no ‘adjusting’ of messages or transactions at the hub.

A decentralized system can resolve some of the drawbacks of the network and centralized system approaches. A decentralized approach can use an overarching blockchain, distributed ledger, or interconnected ledgers and easily enables microgrids running on different blockchains to connect (Li et al. 2017). These methods support a high level of transparency where all participants have access to the original data and can audit to see if it has been updated or tampered with (Saurabh et al. 2020). The drawback of this model is that transparency is balanced against needed levels of privacy.

A VC approach combines features of the three approaches above. Like the network approach, systems participate as a peer and benefit from the features of a network approach, including transparency and protection against a single point of failure. Like a centralized approach, participating systems can reach out to a higher system to discover the standards and identification information which helps to maintain a synchronized system and reliable source of truth. Like a decentralized approach, a repository of standards is stored on a decentralized ledger, which enables traceability and auditability capabilities. However, unlike the decentralized approach, the VC approach does not use a meta-system blockchain to hold the current state of the network or needed information for transactions. Instead, each peer system is a source of truth and can transact directly with other peers.

Given the capabilities of a VC-based approach, we propose a VC-based architecture to address the requirements set forth in Section 3. In the following section, we define the components of our architecture and how they satisfy the requirements identified for a decentralized renewable energy smart grid. The architectural components include VCs and DIDs, defined in Section 2.2, as well as adapters, agents, a metadata blockchain, and governance, defined below.

*Agent*: The agents are the entry point to the system and handle P2P communication between systems. Agents use the metadata blockchain to discover endpoints and prove the source and destination systems.

*Metadata blockchain*: The metadata blockchain is used to store the following: (1) network definitions (e.g., grammars, glossary of terms, data format specifications, semantics, and data model standards); (2) public DID management items (e.g., identifier mapping, system discovery, and public identity endpoints); (3) governance items (e.g., policies, protocols, governance methodology, platform administration mechanisms, maintenance, and sunsetting); (4) the schema credential registry (i.e., VC structure schema). Each participating system needs to run a node that stores a copy of the metadata blockchain.

*Adapter*: Our approach requires an adapter service for system integration, which is a combination of smart contract and application code. An adapter service holds custom logic for communicating and integrating with the system it represents. The adapter performs the following: (1) handles the translation of architecture standards; (2) works with the agent to create the VCs; (3) works with the agent to translate identifiers from local to system-wide and vice versa; (4) uses the agent to query standards and identity information from the metadata blockchain; (5) verifies the credential payload.

*Governance*: There needs to be predictability in how the system will manage interactions in a network from a technical and managerial standpoint. Governance is necessary to manage the network, including the network standards, credential structure schemas, and identifier mapping. Governance is also necessary to define how conflict, upgrades, and policies will be handled. The governance model should use voting mechanisms to implement or amend the metadata blockchain. The exact governance structure must be determined, including how voting will work and which parties will participate.

**Figure 2:**
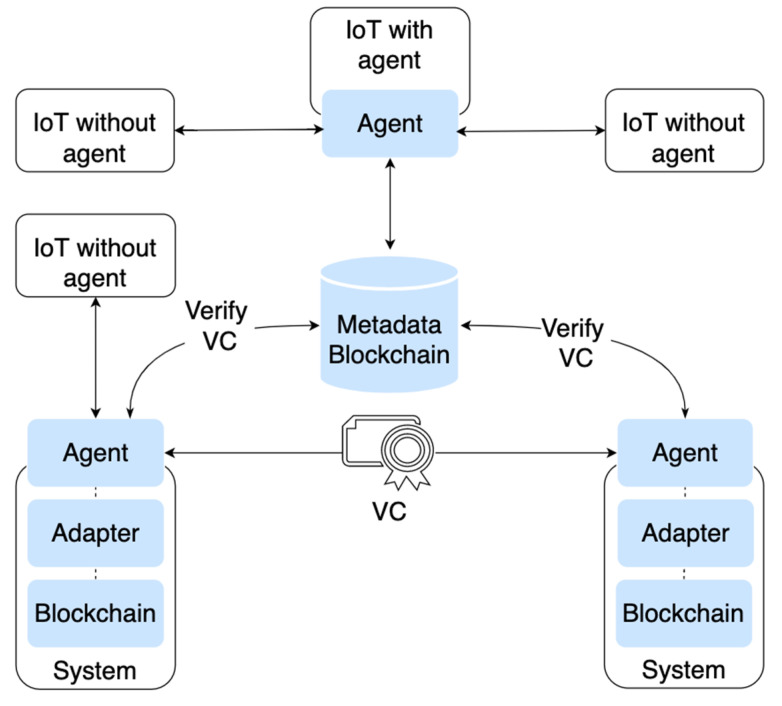
Decentralized Communication in a VC-Centric System.

Figure 2, below, shows how IoT devices, agents, adapters, blockchains and the metadata blockchain can communicate in a VC-centric system.

Below, Table 1 provides detailed architecture requirements in the left-hand column, while the right-hand column defines how these requirements can be met with our proposed VC-based architecture.

### Potential Drawbacks

We have identified the following potential drawbacks to our proposed architecture. First, we suspect that the difference of trust in the underlying data of blockchain and centralized systems will become a significant issue. Blockchain data is immutable, and all external references will continue to be resolvable throughout the lifecycle of the blockchain system. Centralized systems based on databases do not provide that guarantee.

Second, blockchain systems that use smart contracts that run on virtual machines may limit the amount of processing power that a smart contract can access. Limited processing power may limit the ability of smart contracts to generate VCs.

Third, smart contracts ensure that the trust of the blockchain is captured at the time of the credential creation. If code external to the blockchain, such as adapter code using an SDK, has to build the credential, then the trust inherent to the blockchain cannot be fully extended to the credential.

Fourth, the VC approach does not have a consensus mechanism to ensure that transactions are the same between systems. Fifth, VCs with encrypted data could be harvested and exposed when quantum systems that can break existing cryptography methods arrive. Further research should include building a proof-of-concept to test the proposed architecture.

## 5. Conclusions

There are increasing sources of renewable energy that are heterogeneous and geographically dispersed. A resulting shift towards distributed architectures more suitable to manage the decentralized nature of the grid can be observed. The rise of P2P energy trading and microgrids has particularly accelerated this shift.

However, traditional ICTs struggle to balance the increasingly distributed nature of the grid. A main concern is that microgrids and P2P trading networks are presently unable to consistently connect to the main grid, a necessary factor in providing secure and reliable power availability. Blockchain technology has the ability to overcome this challenge and can effectively integrate distributed energy sources into the main grid, including low-voltage energy providers.

In this paper, we contribute a review of the present and future state of decentralized renewable energy grid management based on six technology layers: (1) grid architecture, (2) registries, (3) grid management, (4) privacy, (5) billing, and (6) interoperability. In addition to our review, we contribute a set of blockchain-based requirements to meet the needs of the changing renewable energy market. We additionally contribute a system architecture to meet those needs. After reviewing the existing distributed architecture approaches, we find the most suitable is to take a hybrid approach consisting of a network of distributed blockchain-enabled microgrids connected to the main grid. This architecture best supports secure energy distribution and the systems and processes’ different speed and security demands. Using blockchain technology enables the effective integration of microgrids and enables P2P transactions to support a reliable main power supply.

On a technical level, our blockchain-based architecture uses smart contracts, DIDs and VCs. Smart contracts support automated trading platforms that take real-time demand and availability fluctuations into account. They also have the potential to increase the flexibility of renewable energy markets. Increased flexibility in renewable energy markets can increase consumer mobility and thus increase competition amongst energy providers. Exposing consumers to the dynamic costs in the energy market could also influence more sustainable energy consumption. DIDs and VCs enable peer nodes to communicate and transact directly and enable privacy capabilities. DIDs and VCs use private information to prove something verifiable using information stored in a public registry. They enable subjects to exchange credentials to assert a truth about something without using third parties or disclosing the actual data itself. The VCs can also move data and guarantee that the data was not tampered with during transmission.

There are still several gaps in our and other proposed decentralized renewable energy architectures. First, technical solutions will need to consider the privacy implications of storing information in an immutable ledger and have solutions for involuntary or accidental disclosure of sensitive information. Second, scalability is a barrier, especially concerning discoverability, propagation delays, and time to finality. Third, solutions must be energy efficient to align with the overarching mission of renewable energies. Fourth, a decentralized energy grid’s governance and microgrid boundaries must also be determined to ensure grid quality and reliability. Fifth, an overarching gap affecting all aspects of the network is interoperability. Without interoperability, the solutions are siloed, will not reach their full potential, and will prevent the development of a seamless system with pluggable solutions.

## Figures and Tables

**Table 1:** Architecture requirements and the VC approach.

Architecture Requirements		VC Handling of Requirements
**Framework Requirements**		
1. Framework identity requirements		
a.	Supported universal DIDs for organizations, users, and digital assets.	VCs will use DIDs to identify the issuers, recipients, and payload data and use DIDs for assets, tasks, and transactions.
b.	Standards that map multiple blockchains and centralized system-specific identities to the DID that uniquely represents a digital asset across various systems.	VCs will have a DID method specification that identifies a DID across systems. The adapter and agent for each system will be responsible for the system-specific mapping information in the DID method-specific data.
2. Framework security and privacy requirements		
a.	Mechanisms for private or confidential transactions between different systems.	Using P2P communication with encrypted VC payloads ensures private transactions.
b.	Mechanisms for maintaining transaction privacy in cross-system transactions.	Using P2P communication with encrypted VC payloads ensures private transactions.
3. Framework interoperability requirements		
a.	Defined standards for connecting with external systems.	Agents are responsible for connecting with other systems. Agent code is the same for each system.
b.	Defined standards for integrating with IoT devices.	IoT devices can either (i) run an agent on the device, (ii) connect to an agent on another device, or (iii) connect to an agent in the cloud through its adapter.
c.	Defined standards for connecting with external blockchains.	Blockchains can connect to the system through their adapter and connect to other systems via the agent. The adapter can connect to the blockchain using SDK and smart contracts.
4. Framework data and processes requirements		
a.	A standard protocol for the exchange of data between systems.	The metadata blockchain will hold the VC schema, the data model, and data mapping information.
b.	A standard protocol for orchestrating tasks, actions, and logic between subsystems.	The metadata blockchain will hold the VC schema, the data model, and data mapping information.
c.	A standard mechanism for performing analytics or reporting on data stored across multiple subsystems while preserving applicable data privacy.	Each system is the single source of truth for the data it holds. Analytics and reporting need to contact each system using the identifiers in the metadata blockchain to build the report data. Each system already holds access rules that apply to any cross-system queries.
d.	Monitored external data sources, monitored for their health.	Mediators can handle situations where an object is unavailable, e.g., an IoT device using mobile communication. The mediator can store and forward the communication of VCs.
5. Framework architecture requirements		
a.	Support public and permissioned blockchain deployment models.	Adapters for each system will account for the specifics of the blockchain deployment model.
b.	Include a discovery service to discover endpoints for participating systems.	Metadata blockchain will provide discovery information that the agents can use to help the adapters correctly address the data issuer or recipient using fully qualified DIDs.
c.	Discovery services that implement health checks for endpoint management and failover.	Mediators can store and forward VC communication to handle situations where a system is unavailable or an endpoint changes.
d.	A discovery service that seamlessly facilitates adding, removing, or modifying endpoints.	Metadata blockchain will provide discovery information that agents can use to help the adapters correctly address the data issuer or recipient using fully qualified DIDs.
e.	Platform infrastructure that supports on-premise or cloud deployment options without vendor lock-in.	Participating system adapters will account for the specifics of the cloud deployment and can be customized to work with any cloud platform.
f.	A set of standard interfaces for external systems to accomplish specific business functions.	The metadata blockchain will hold the schemas that can standardize business communication.
g.	Supported asynchronous communication with appropriate message queuing.	Mediators can store and forward VC communication to handle situations where a system is unavailable or an endpoint changes.
h.	Notification capabilities across systems.	VCs can support any type of data. The agents can facilitate P2P direct messaging, enabling notifications that do not require attributions or verification.
**Platform**		
1. Platform identity requirements		
a.	Queries that enable an organizational management service to map a DID to a specific organization.	The metadata blockchain will hold the public DIDs in a DID registry.
b.	Unique identifiers that are persistent on an organization’s platform.	DIDs contain a system-specific component. Even if two systems used identical representations of an object, the system identification data would enable universally unique IDs.
c.	Digital assets with unique identifiers that are persistent on their organization’s platform.	
*Platform interoperability requirements*		
a.	A standardized messaging layer that connects with IoT devices, including support for IoT events and selective data querying.	VCs can support any type of data, including messaging data. The adapter for the IoT device and the DID method-specific identity information can handle IoT events and selective querying.
b.	A standardized messaging layer that connects with traditional IT systems.	VCs can support any type of data, including messaging data.
c.	A standardized messaging layer that connects with external blockchains.	
2. Platform security and privacy requirements		
a.	A mechanism for enabling private transactions.	Using P2P communication with encrypted VC payloads ensures that a transaction is private.
b.	No exposure of user private cryptographic materials to the overarching system or individual system administrators.	Each user or organization will sign their VCs with their private keys; the system will not require private keys.
c.	No PII may be committed to the ledger.	The metadata blockchain only holds schemas, public endpoints, and public keys; it does not hold the PII of individuals.
3. Platform governance requirements		
a.	User actions that are auditable, comprehensive, and immutable.	The VC approach does not create new actions beyond the credential’s creation, delivery, and receipt; additional, auditable actions can be added by customizing the adapter.
b.	All actions and data traceable to the organization/user that published the data or executed the action.	DIDs will be used in all cross-system communications and traceability.
4. Platform analytics requirements		
a.	A standard mechanism for real-time data analysis within the blockchain without violating the data privacy controls or exposing private data to system administrators.	The VC approach does not keep a shadow copy of the data, so it does not need a duplicate set of privacy controls.
**Use case-specific**		
1. Use case-specific, functional requirements		
a.	A standard mechanism to define, store, and exchange asset lifecycle information in an immutable data structure.	The metadata blockchain will hold the asset standards that the adapter will use to translate those standards to the specific platform.
b.	Supports storing asset lifecycle events and data in real-time.	The asset stays on the original system; a copy is not maintained. There is no performance lag on a copy.
c.	Supports storage of asset lifecycle events and data in an immutable fashion.	The digital asset depends on the implementation of the original system that stores it. A VC does not affect the abilities of the original system.
d.	An identifier that is unique across systems for each asset.	The fully qualified DID gives a universally unique ID to all assets based on the asset ID generation of the underlying system and the identification of the system itself.
e.	Asset data that includes physical origin, digital origin, and asset composition information. This information may be gathered from and communicated to other systems.	A VC can hold the complete data on an asset and can be passed between systems. It is up to the receiving system implementation on how much of that data they wish to retain.
f.	Supports tracking of parent–child asset relationships, including scenarios where the parent and child assets are tracked in different systems.	The schema of the VC payload can have hierarchical representations, and a credential can also contain other credentials. The DIDs are references to data in systems, so the relationship can be represented without copying the data.
g.	Be configurable to allow for nuanced business requirements of specific industry verticals. Configuration must include asset attributes, asset events and event attributes, and asset and event validation rules. The configuration may consist of rules of origin and tariff rules.	The adapter and the metadata blockchain can store the industry-specific elements of the system.
h.	Asset division and assembly support, e.g., division of batches or runs, plates or coils, and support for assemblies, like ingredients for a batch or a combination of products to construct a new one, like a car.	DIDs have a method-specific data element that can be configured in the metadata blockchain to help identify the origins of divisions and component assemblies. VCs can also be kept and used to verify things.
2. Use case-specific privacy and security requirements		
a.	Data access on a least-privilege basis.	Access to the data is specific to the system itself. The system-specific adapter controls the local data access for locally building a VC or handling a request from an external party.
b.	Organization registration and onboarding based on an organization’s relationship with a specific digital asset. User registration and onboarding must be based on role-based access control (RBAC).	VCs can be used to onboard users, organizations, and devices. The credential is a machine-readable way to determine if the subject fits a role.
3. Use case-specific interoperability requirements		
a.	Reliable, integrated external system providers.	Adapters are required for each service that participates in the entire system.
b.	Sanitized and validated inputs that are then committed to a network distributed ledger.	Each adapter is in charge of translating between systems using the metadata blockchain standards and data model.
c.	Real-time analytics that supports reporting, AI, or other analysis tools.	The handshaking required for VC communication may preclude real-time reporting. There will always be a lag, a trade-off of having verifiability.
4. Use case-specific, non-functional		
a.	Dependence on the platform’s identity and discovery services, but support for external shared services.	The adapter and agent of the platform will handle the discovery of external services for the underlying system.
b.	Compliance with NIST.	A NIST audit will be required to ensure compliance. No significant impediments to NIST compliance are foreseen.
